# First molecular detection of *Rickettsia africae* in ticks from the Union of the Comoros

**DOI:** 10.1186/1756-3305-7-444

**Published:** 2014-09-22

**Authors:** Amina Yssouf, Cristina Socolovschi, Tahar Kernif, Sarah Temmam, Erwan Lagadec, Pablo Tortosa, Philippe Parola

**Affiliations:** Unité de Recherche en Maladies Infectieuses et Tropicales Emergentes (URMITE), UM63, CNRS 7278, IRD 198, Inserm 1095, WHO Collaborative Center for Rickettsioses and Other Arthropod-borne Bacterial Diseases, Faculté de Médecine, Aix Marseille Université, 27 bd Jean Moulin, 13385 Marseille cedex 5, France; Centre de Recherche et de Veille sur les Maladies Emergentes dans l’Océan Indien (CRVOI)-Plateforme de Recherche CYROI, 2, rue Maxime Rivière, 97490 Ste Clotilde, La Reunion, France; Université de La Réunion, Ste Clotilde, La Réunion, France

**Keywords:** Cattle ticks, *Rickettsia africae*, *Amblyomma variegatum*, *Rhipicephalus*, Comoros

## Abstract

**Background:**

*Rickettsia africae* is the agent of African tick bite fever, a disease transmitted by ticks in sub-Saharan Africa. In Union of the Comoros, a recent study reported the presence of a *Rickettsia africae* vector but no information has been provided on the circulation of the pathogenic agent in this country.

**Methods:**

To evaluate the possible circulation of *Rickettsia* spp. in Comorian cattle, genomic DNA was extracted from 512 ticks collected either in the Union of the Comoros or from animals imported from Tanzania and subsequently tested for *Rickettsia* infection by quantitative PCR.

**Results:**

*Rickettsia africae* was detected in 90% (60/67) of *Amblyomma variegatum*, 1% (1/92) of *Rhipicephalus appendiculatus* and 2.7% (8/296) of *Rhipicephalus* (*Boophilus*) *microplus* ticks collected in the Union of the Comoros, as well as in 77.14% (27/35) of *Amblyomma variegatum* ticks collected from imported cattle. Partial sequences of both bacterial *gltA* and *ompA* genes were used in a phylogenetic analysis revealing the presence of several haplotypes, all included within the *Rickettsia africae* clade.

**Conclusions:**

Our study reports the first evidence of *Rickettsia africae* in ticks collected from the Union of the Comoros. The data show a significant difference of infection rate of *Rickettsia africae* infected ticks between the Islands, with maximum rates measured in Grande Comore Island, sheltering the main entry port for live animal importation from Tanzania. The high infection levels reported herein indicate the need for an in-depth assessment of the burden of rickettsioses in the Union of the Comoros, especially among those at risk of infection, such as cattle herders.

## Background

Tick-borne rickettsioses are considered among the oldest known vector-borne zoonotic diseases; they are caused by obligate intracellular Gram-negative bacteria belonging to the spotted fever group (FSG) of the genus *Rickettsia*[1]. Many species of this genus are considered to be vertically transmitted symbionts of invertebrates, suggesting that the arthropod vectors act as reservoirs or amplifiers of rickettsiae in the wild [2–4]. In sub-Saharan Africa, several rickettsial strains have been isolated and detected from ticks and vertebrate animals [5], among which *Rickettsia africae*, the etiological agent of African tick-bite fever (ATBF), is the most common [3, 6, 7]. The main tick-vectors of *Rickettsia africae* are *Amblyomma hebraeum* in southern Africa and *Amblyomma variegatum* in West, Central and Eastern Africa, as well as in the eastern Caribbean [7–11]. In the Indian Ocean, *Rickettsia africae* has been previously detected in *Amblyomma variegatum* in La Reunion and Madagascar [4, 12] but never in arthropod and human samples from other islands of the region, including the Comorian Islands.

The Union of the Comoros is composed of three volcanic islands: Grande Comore (the youngest and most elevated island), Anjouan and Moheli. The archipelago is located in the western Indian Ocean, at the northern entrance of the Mozambique Channel between Madagascar and the East African coast, and is characterized by a warm and humid tropical climate. Cattle are imported as a food source, mainly from Madagascar. In 2000, a free trade agreement was signed between Comoros and Tanzania, facilitating the reciprocal travel and flow of cattle. Consequently, the export of cattle from Tanzania to Comoros, and particularly to Grande Comore Island, has steadily increased [13]. The likelihood of an increase in the number of pathogens introduced through cattle-associated ticks has also risen.

Recently, an entomological survey carried out on all three islands of the Union of the Comoros showed the presence of *Amblyomma variegatum*, *Rhipicephalus appendiculatus* and *Rhipicephalus microplus* on cattle [13]. Thus, the aim of this study was to detect and determine the prevalence of *Rickettsia* species that infect ticks on autochthonous cattle and on cattle imported from Tanzania and to evaluate the role of cattle importation in the introduction and of the human pathogen, *Rickettsia*, throughout the country.

## Methods

### Study sites and tick sampling

The present study used adult ticks that were previously collected to describe cattle tick diversity and distribution in the country. Briefly, the ticks were collected from animals on the three islands of the Union of the Comoros, including 16 of the 17 districts of the country. Adult ticks were collected on cows and goats from the three islands during the 2010 rainy season. For each district, between one and five animals were sampled and identified, and the number of collected ticks was recorded for each site of collection. Following the same protocol, ticks were also collected from cattle imported from Tanzania that were held in the quarantine enclosure located in the harbor vicinity, or in any of the three other quarantine enclosures located in the capital, Moroni (Grande Comore). All ticks were immediately stored in 70% ethanol until morphological and molecular analyses. Tick species were determined morphologically using standard identification keys [14].

### DNA extraction and PCR detection of *Rickettsia*

Each tick was sliced longitudinally with a disposable scalpel, and each half was crushed in a buffered solution (G2) with proteinase K (Qiagen Hilden, Germany) and incubated at 56°C overnight. Total DNA from half of each tick was extracted in 50 μl of eluate using the EZ1 DNA Tissue kit (Qiagen, Hilden, Germany). Rickettsial DNA detection was performed by quantitative PCR using the Eurogentec MasterMix Probe PCR kit (Qiagen, Hilden, Germany) following the manufacturer’s instructions with a final volume of 10 μl in each reaction as previously described [15]. Each DNA sample was tested by quantitative (q) real-time PCR using a CFX 96 Real Time System (BIO-RAD, Singapore). The presence of SFG *Rickettsia* from was determined with a Taqman probe (Eurogentec, Seraing, Germany) and RKND03F and RKND03R specific primers targeting the citrate synthase A (*glt*A) encoding gene [16, 17]. Positive samples were subsequently screened with a previously described *Rickettsia africae*-specific qPCR method. Samples with fewer than 35 cycle thresholds (Ct) were considered positive [18].

In order to generate sequence data allowing phylogenetic analyses of infecting *Rickettsia*, *gltA* and *ompA* were amplified and subsequently sequenced from a subset of randomly selected *Rickettsia*-positive tick samples. A fragment of *gltA* gene was amplified using the Rp CS.409d and CS.1258n primers, previously reported to amplify a 750-bp fragment from all known *Rickettsia* species [6], and *ompA* gene was targeted by using the primers 190.70,190.180, and 190.701, amplifying a 629–632-bp fragment of SFG *Rickettsia*[2, 6].

For each PCR reaction, one positive control (*Rickettsia montanensis* DNA) and 2 negative controls (sterile water containing DNA extracted from uninfected ticks maintained in laboratory colonies) were included, with the exception of the *Rickettsia africae*-specific qPCR reaction in which *Rickettsia africae* DNA was used as positive control.

The resulting PCR products were purified and directly sequenced using a BigDye Terminator Cycle Sequencing Kit (Perkin Elmer Applied Biosystems, USA) and an ABI PRISM automated sequencer (Applied Biosystems, USA), as previously described [6].

### Sequence editing and phylogenetic analyses

Sequences were analyzed using Chromas Pro (version 1.49 beta Technelysium Pty Ltd, Tewantin, Australia) and compared with sequences available in the GenBank database using NCBI BLAST. Multiple sequence alignments were noted at the nucleotide and amino acid levels using ClustalW implemented in BioEdit software. The sequences matched with sequences from other *gltA* and *ompA* rickettsiae retrieved from the GenBank database. *Rickettsiaconorii* and *Rickettsia rickettsii* sequences have been chosen as root sequences. Accession numbers of the GenBank sequences used for the genetic analyses are indicated in the phylogenetic trees. The selection of the DNA substitution model that best fit the data was performed with MEGA 5.2 and was considered for phylogenetic analyses. We selected different models of nucleotide substitution using the corrected Akaike information criterion. Bayesian phylogenetic inference (BI) was carried out using Mr Bayes 3.1.2 [19] with two independent runs of four incrementally heated, Metropolis Coupled Markov chain Monte Carlo (MC) inquiries beginning with a random tree. MC queries were run for 1,000,000 generations with trees and associated model parameters sampled every 200 generations. The initial 1000 trees in each run were discarded as burn-in samples, and the harmonic mean of the likelihood was calculated by combining each of the two independent runs.

### Statistical analysis

A statistical analysis was performed to compare the prevalence of *Rickettsia africae* between the islands using the Mantel-Haenszel test implemented in Epi-info version 3.5 followed by the Yates correction option. Differences were considered statistically significant for P values <0.05.

## Results

### PCR detection of *rickettsia*

#### Ticks collected on autochthonous cattle

**Figure 1:**
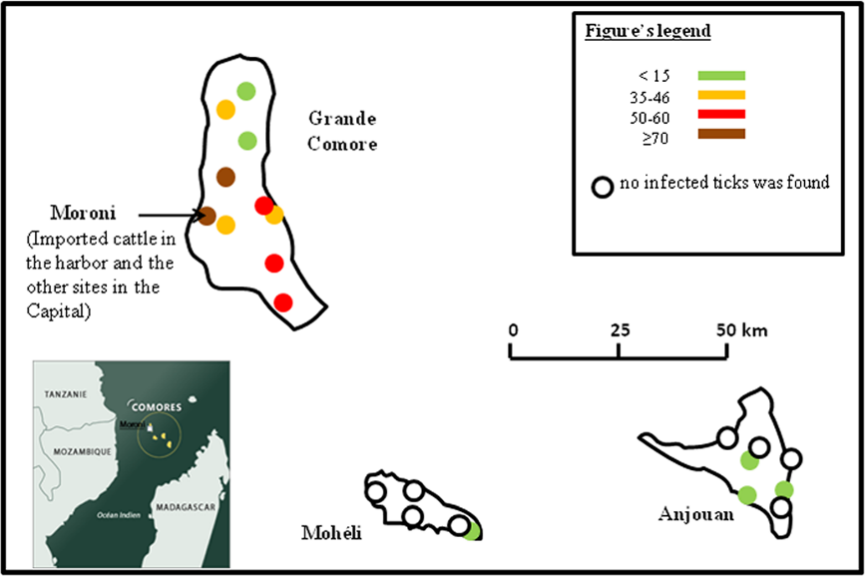
**Prevalence of infected ticks by island and regions.**

Rickettsial DNA was detected in 14% (67/477) of ticks from the Union of the Comoros. Table 1 presents the distribution of samples positive for *Rickettsia* by tick species and location. The mean Ct ± SD value of *glt*A amplification by qPCR of positive tick samples was 28.34 ± 3.11. All positive samples were also positive for an *Rickettsia africae*-specific qPCR with a mean Ct ± SD value of 28.51 ± 2.03. On Grande Comore Island, 90% (60/67) of *Amblyomma variegatum* ticks tested positive for *Rickettsia africae*, with Ct values averaging 28.67 ± 1.95, while 1.9% (2/105) of *Rhipicephalus* spp. ticks were positive, with a Ct averaging 28.88 ± 1.8 (Figure 1). The prevalence of *Rickettsia africae* on Anjouan Island was 8.12% (13/160), and a significant difference in prevalence was observed between Anjouan and Grande Comore 32.48% (51/157) (Mantel-Haenszel test, P value < 0.001). Among the positive samples from Anjouan, 61.5% (8/13) were obtained from *Amblyomma variegatum* ticks, while the remainders were from *Rhipicephalus microplus* (Table 1). In Mohéli, one of the five regions we visited had cattle carrying infected ticks (Figure 1). The prevalence of *Rickettsia africae* on this island was 1.9% (3/160), which was also significantly different from that found on Grande Comore (Mantel-Haenszel test, P-value <0.001) and Anjouan (Mantel-Haenszel test, P-value = 0.016). All positive ticks from Mohéli were *Amblyomma variegatum* (Table 1), with positive PCRs displaying an average Ct of 25.24 ± 1.97.

**Table 1:** **Prevalence of** ***Rickettsia africae*** **by species and regions**

Species	***Amblyomma variegatum***	***Rhipicephalus microplus***	***Rhipicephalus appendiculatus***	Number of ticks tested by region
Regions				
Grande Comore	94.23% (49/52)	8% (1/13)	1% (1/92)	157
Anjouan	89% (8/9)	3.3% (5/151)	-	160
Moheli	7% (3/28)	0% (0/132)	-	160
Ticks collected from imported cattle	77.14% (27/35)	-	-	35
Total from the Union of the Comoros	65.17% (58/89)	2.7% (8/296)	1% (1/95)	477

The amplification, sequencing and BLAST analyses of *ompA* and *gltA* genes from positive DNA samples extracted from *Amblyomma variegatum*, *Rhipicephalus microplus* and *Rhipicephalus appendiculatus* ticks confirmed the presence of *Rickettsia africae* in 14% of our samples. The sequence analysis of *ompA* genes obtained from *Rhipicephalus* spp. samples revealed 99.0 to 99.6% nucleotide identity with *Rickettsia africae* detected from *Rhipicephalus evertsi evertsi* in Senegal (GenBank accession numbers JN043509), while *R. africae* sequenced from *Amblyomma variegatum* showed 98 to 99.7% average identity with *R. africae* detected in Ethiopia from the same tick species (GenBank accession numbers CP001612). The sequencing of *gltA* genes obtained from *Amblyomma variegatum* and *Rhipicephalus* spp. showed 99.1 to 99.42% average identity with the published sequences of *Rhipicephalus africae* that were amplified from *Amblyomma variegatum* collected in the West Indies (GenBank accession number HM050288).

#### Ticks collected from imported cattle

Of the ticks collected from cattle imported from Tanzania, 77.14% (27/35) were positive for *Rickettsia* spp., with a Ct average of 27.75 ± 4.13. All of the samples detected positive for *Rickettsia* spp. were also positive for *Rickettsia africae* by qPCR (Figure 1), with Ct averages of 26.7 ± 4.67.

The sequencing of *ompA* genes obtained from *Amblyomma variegatum* collected from imported cattle showed an average of 98% alignment with published sequences of *Rickettsia africae* from *Amblyomma variegatum* collected in Antigua (GenBank accession number EU622980).

The sequence analysis of the *gltA* gene obtained from *Amblyomma variegatum* ticks revealed 98.0 to 99.0% nucleotide identity with the *gltA* gene from *Rickettsia africae* detected in *Amblyomma variegatum* from Ethiopia (GenBank accession numbers CP001612) and in *Rhipicephalus evertsi evertsi* from Senegal (GenBank accession numbers HM050288), respectively.

### Phylogenetic analysis

**Figure 2:**
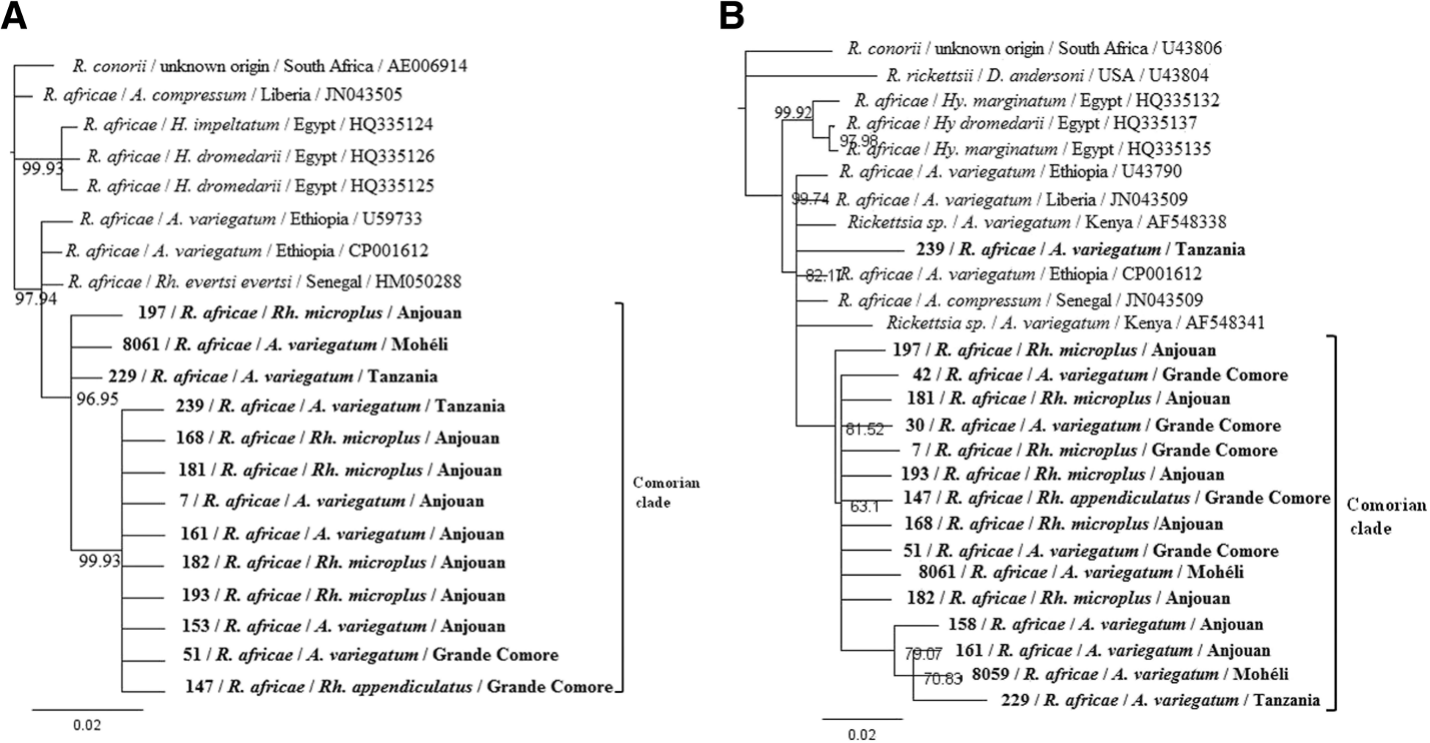
**Phylogenetic tree inferred from the comparison of** ***Rickettsia africae*** **strains from specimens tested and selected GenBank** ***Rickettsia africae*** **sequences.** Posterior probabilities are expressed in percentages and indicated at branch nodes. The sequences generated in this study are highlighted in bold. **A**: Phylogenetic tree based on the *gltA* citrate synthase-encoding gene. **B**: Phylogenetic tree based on the *ompA* outer membrane protein A gene. Abbreviation: *Rh* = *Rhipicephalus*; *Hy* = *Hyalomma*; *D* = *Dermacentor.*

The best DNA substitution model fitting the data was determined to be HKY for both the *gltA* and *ompA* sequences. The Bayesian Inference tree based on *gltA* sequences (Figure 2A) showed a distinct phylogenetic Comorian clade (posterior probability > 0.968) in which one well-supported group of sequences (posterior probability > 0.999) included sequences from *Rickettsia africae* infecting ticks sampled in Grande Comore and Anjouan but not Mohéli, and a *Rickettsia africae* sequence obtained from a tick imported from Tanzania. The Bayesian Inference tree based on *ompA* sequences (Figure 2B) showed a similar tree topology, which consolidate our analysis.

Consensus sequences of *Rickettsia africae* infecting ticks sampled on Grande Comore, Mohéli, Anjouan and Tanzania were generated and aligned to determine nucleotide mutations specific to a geographic origin. No specific mutation was observed for the *ompA* genes (data not shown); interestingly, the *gltA* sequences originating from Mohéli have 5 specific non-synonymous mutations (Table 2). The same analysis was conducted to construct consensus sequences of *Rickettsia africae* isolated from distinct tick species, but no specific mutation was observed in either the *ompA* or *gltA* gene (data not shown).

**Table 2:** **Non-synonymous mutations in** *Rickettsia* ***africae gltA*** **Mohéli sequences**

Nucleotide position (U59733)	560	566	635	692	1067
Codon sequence	GAT → AAT	CCG → TCG	CCA → GCA	AAT → TAT	AGA → GGA
Amino-acid mutation in Mohéli *gltA* sequence	D → N	P → S	P → A	N → Y	R → G

## Discussion

*A. variegatum* is the main vector of *Rickettsia africae*, a spotted fever group (SFG) *Rickettsia* bacterium in sub-Saharan Africa [9, 20, 21], though it is also considered a competent vector for other human and animal pathogens [22], including the highly virulent Crimean-Congo hemorrhagic fever virus [23]. In the present study, we show for the first time the presence of *Rickettsia africae* in cattle ticks collected from the Union of the Comoros. The confirmation of the presence of this *Rickettsia* spp. provides background for further epidemiologic and clinical investigations of tick-borne diseases in the Union of the Comoros. Indeed, other than *Theileria parva*, a parasitic protozoan that is the causative agent of the East Coast fever in cattle and that was previously detected in *Rhipicephalus appendiculatus*[24], no other tick-borne pathogens have been detected in this country.

We provide evidence for *Rickettsia africae* infection in *Amblyomma variegatum* ticks from all three islands of the Union of the Comoros. Although the *Amblyomma variegatum* tick infection rate varied among the islands as observed in Grande Comore and Anjouan, further study needs to confirm this result. The presence of *Rickettsia africae*-infected ticks in Grande Comore and Anjouan is congruent with the geographic distribution of *Amblyomma variegatum*[8]. The high prevalence of *Rickettsia africae* (90%) in *Amblyomma variegatum* collected in all study sites of Grande Comore showed endemicity of this bacterium and that this tick species is the reservoir of *R. africae* in the Archipelago. Ticks of the genus *Amblyomma* are considered to be the main vectors for *Rickettsia africae* although this bacterium has recently been found infecting other genera, including *Rhipicephalus*[21, 22, 25]. In this study, we also found *Rhipicephalus* spp. ticks carrying *Rickettsia africae* DNA although the presence rate was substantially lower (2%) than the rate measured in *Amblyomma variegatum* (65.17%). However, our data do not provide direct evidence for the vector competence of the *Rhipicephalus* (*Boophilus*) *microplus* genus for *Rickettsia africae* because *Rhipicephalus*-positive ticks were always collected on animals infested with *A. variegatum* ticks that tested positive for *R. africae.* Thus, we can hypothesize that *Rhipicephalus* ticks that were positive for *Rickettsia africae* acquired the pathogen during a blood meal, but this does not prove that these species of ticks are competent vectors for *Rickettsia africae*. Further experimental evaluations of vector competence clearly need to be carried out in order to establish the vector competence of *Rhipicephalus* ticks.

Ticks sampled on cattle imported from Tanzania showed a prevalence of 77.14% for *Rickettsia africae*, which is coherent with serological studies previously carried out in Tanzania that also showed high seroprevalence levels [26]. *Rickettsia africae* polymorphism did not show any island or tick host species structuration. Comorian haplotypes were closely related with African haplotypes, thus strengthening the hypothesis of an African origin for *Rickettsia africae* in the Comoros archipelago. Phylogenetic trees could not determine the origin of *Rickettsia africae* infection in the ticks imported from Tanzanian cattle. However, the polymorphism of *omp*A and *glt*A is notoriously weak and sequencing of additional markers may help in resolving this issue. Citrate synthase is a component of nearly all living cells and is one enzyme of the citric acid cycle, a key metabolic pathway that plays a key role in energy production [27]. The *OmpA* gene plays a role in a protective immune response and is considered as a good candidate for phylogenetic analysis for most of the SFG *Rickettsiae*[2]. These gene sequences showed no clear differences between east African and Comorian bacterial haplotypes, thus supporting an African/Comorian *Rickettsia africae* metapopulation, although the use of additional markers, or the full sequencing of bacterial isolates would obviously provide more robust information.

## Conclusion

The PCR assays and sequence analyses provide new information on the epidemiology of ticks infected with *Rickettsia africae* in the Union of the Comoros. The detection of *Rickettsia africae* in ticks collected from live cattle imported from Tanzania represents a risk to local farms. Our results strengthen the need for an evaluation of the burden of ATBF in the human populations in contact with cattle. Together with the previous investigation reporting the introduction of a tick species of veterinary importance new to the country, the present study clearly shows that because of the insularity, ticks and parasites introduction into the country represents a risk of biological invasion. Therefore, increased vigilance is required to limit this risk [28].
